# Extracellular vimentin is sufficient to promote cell attachment, spreading, and motility by a mechanism involving N-acetyl glucosamine-containing structures

**DOI:** 10.1016/j.jbc.2023.104963

**Published:** 2023-06-24

**Authors:** Robert Bucki, Daniel V. Iwamoto, Xuechen Shi, Katherine E. Kerr, Fitzroy J. Byfield, Łukasz Suprewicz, Karol Skłodowski, Julian Sutaria, Paweł Misiak, Agnieszka Z. Wilczewska, Sekar Ramachandran, Aaron Wolfe, Minh-Tri Ho Thanh, Eli Whalen, Alison E. Patteson, Paul A. Janmey

**Affiliations:** 1 Department of Physiology, Institute for Medicine and Engineering, University of Pennsylvania, Philadelphia, Pennsylvania, USA; 2 Department of Medical Microbiology and Nanobiomedical Engineering, Medical University of Białystok, Białystok, Poland; 3 Faculty of Chemistry, University of Białystok, Białystok, Poland; 4 Ichor Life Sciences, Inc, LaFayette, New York, USA; 5 Lewis School of Health Sciences, Clarkson University, Potsdam, New York, USA; 6 Physics Department, BioInspired Institute, Syracuse University, Syracuse, New York, USA

**Keywords:** vimentin, cytoskeleton, extracellular matrix, cell adhesion, cell migration, glycocalyx

## Abstract

Vimentin intermediate filaments form part of the cytoskeleton of mesenchymal cells, but under pathological conditions often associated with inflammation, vimentin filaments depolymerize as the result of phosphorylation or citrullination, and vimentin oligomers are secreted or released into the extracellular environment. In the extracellular space, vimentin can bind surfaces of cells and the extracellular matrix, and the interaction between extracellular vimentin and cells can trigger changes in cellular functions, such as activation of fibroblasts to a fibrotic phenotype. The mechanism by which extracellular vimentin binds external cell membranes and whether vimentin alone can act as an adhesive anchor for cells is largely uncharacterized. Here, we show that various cell types (normal and vimentin null fibroblasts, mesenchymal stem cells, and A549 lung carcinoma cells) attach to and spread on polyacrylamide hydrogel substrates covalently linked to vimentin. Using traction force microscopy and spheroid expansion assays, we characterize how different cell types respond to extracellular vimentin. Cell attachment to and spreading on vimentin-coated surfaces is inhibited by hyaluronic acid degrading enzymes, hyaluronic acid synthase inhibitors, soluble heparin or N-acetyl glucosamine, all of which are treatments that have little or no effect on the same cell types binding to collagen-coated hydrogels. These studies highlight the effectiveness of substrate-bound vimentin as a ligand for cells and suggest that carbohydrate structures, including the glycocalyx and glycosylated cell surface proteins that contain N-acetyl glucosamine, form a novel class of adhesion receptors for extracellular vimentin that can either directly support cell adhesion to a substrate or fine-tune the glycocalyx adhesive properties.

Uncontrolled release of intracellular constituents to the extracellular space is generally detrimental to an organism. Potent mechanisms have developed to depolymerize and clear large cytoskeletal filaments such as F-actin from the bloodstream where they otherwise have damaging or proinflammatory effects on vasculature and other organs (1, 2, 3). Perhaps because of the chemical instability of microtubules in GTP-devoid extracellular fluids, no such scavenging system has been described for tubulin, although release of microtubule-binding proteins such as tau is associated with neurodegenerative disease (4, 5, 6, 7). Intermediate filaments (IFs), the most stable element of the cytoskeleton, also do not appear to have active extracellular disassembly and clearance mechanisms, and studies implicate extracellular IF proteins, especially extracellular vimentin, in a range of human diseases (8, 9, 10, 11, 12).

Vimentin is a type III IF protein that forms part of the cytoskeleton of mesenchymal cells, but it can be actively exported to the extracellular environment. This so-called extracellular vimentin has been shown to perform diverse functions in different settings (9, 11, 13, 14). Vimentin's most widely recognized function is that of an intracellular IF cytoskeletal protein that modulates the viscoelastic properties of cells as well as spatially regulating various intracellular signals (15, 16, 17). Despite not having a signal peptide or other features of conventionally secreted proteins, vimentin has now been clearly shown to be selectively released into the extracellular space by active processes, rather than appearing in the extracellular environment solely as the result of mechanical damage to the cell membrane permeability barrier. Extracellular expression of vimentin appears to require at least two active processes (8, 10, 18). First is activation of protein arginine deaminases or protein kinases that either citrullinate or phosphorylate key residues that are required for vimentin to assemble into filaments. Activation of these processes leads to solubilization of vimentin into small oligomeric units. The second process needed for vimentin to be released into the extracellular space or localized to the exterior surface of the cell membrane, involves the unconventional type 3 secretion pathway (19). Once exported to the cell surface or the extracellular space, vimentin can have numerous effects on endogenous host cells or act as an adhesive cofactor for the invasion of specific bacteria or viruses that express on their surface ligands for vimentin (9, 11, 12, 13, 14).

Many sources of extracellular vimentin have been identified. When monocytes are activated to macrophages, they release vimentin to their exterior membrane surface where it enhances clearance of pathogens and apoptotic cells (8, 20, 21). Neutrophils release vimentin as a component of neutrophil extracellular traps (18) often after citrullination of vimentin (22, 23). In wounds to the eye lens that mimic cataract surgery, resident inflammatory cells release vimentin both to their cell surface and to the underlying basement membrane, where it promotes formation of myofibroblasts and a pathologic fibrotic state (24, 25). In some tumors, endothelial cells secrete vimentin by the unconventional type 3 secretory pathway, where extracellular vimentin activates the VEGF receptor to promote angiogenesis (19). Other cell types release vimentin on the external surface of exosomes where it plays multiple roles in wound healing and development (26, 27).

How extracellular vimentin interacts with eukaryotic cell membranes is less well characterized. Several different transmembrane protein complexes have been identified as potential receptors for vimentin, but there is not yet consensus for any specific membrane protein as the unique or most important receptor for vimentin. An alternate hypothesis for how vimentin binds the cell surface is based on its affinity for polysaccharides. A compelling study shows that vimentin binds selectively to polymers bearing multiple copies of N-acetyl glucosamine (GlcNAc) but not most other types of sugar moieties (28). Vimentin also binds glycosaminoglycans, including hyaluronic acid (HA) and heparin, in which one of the two carbohydrate units is either GlcNAc or a closely related structure (29). This affinity for GlcNAc-containing cell surface structures and the fact that many or perhaps all of the proposed vimentin binding cell surface proteins are themselves glycosylated suggest that either the HA- and heparin-rich glycocalyx or transmembrane proteins that are heavily glycosylated are important elements in the mechanism by which extracellular vimentin binds and activates eukaryotic cells.

Whether vimentin in a basement membrane or a substrate is sufficient to promote cell adhesion or whether it primarily acts to modify adhesion to more common extracellular matrix (ECM) ligands such as fibronectin or collagen is also unknown. In this study, we prepare inert hydrogel substrates that bear only covalently attached vimentin as a potential cell ligand and show that multiple cell types bind these surfaces, which promote spreading and motility but not stress fiber formation, large focal adhesion formation, or cell proliferation. Cells bound to vimentin-coated substrates retain mechanosensing properties, but they are different compared to those on collagen- or fibronectin-coated surfaces. Adhesion to vimentin-coated surfaces can be blocked with soluble GlcNAc, and addition of purified vimentin or its citrullinated variant enhances cell migration through three-dimensional (3D) collagen matrices.

## Results

### Adhesion to vimentin-coated gels

**Supporting Figure S1:**
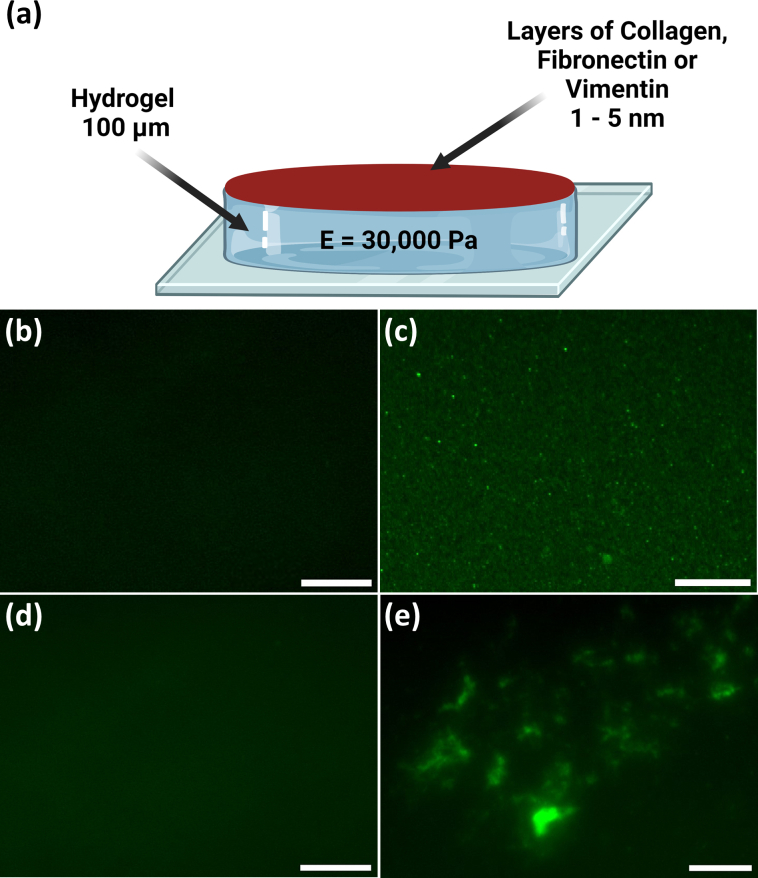
*A*, schematic diagram of polyacrylamide gel coated with different proteins. 30 kPa gel with collagen (*B*) or vimentin (*C*) stained with an anti-vimentin antibody. 30 kPa uncoated (*D*) or collagen-coated (*E*) gel stained with rhodamine-labeled vimentin. Scale bar, 20 μm.

**Supporting Figure S2:**
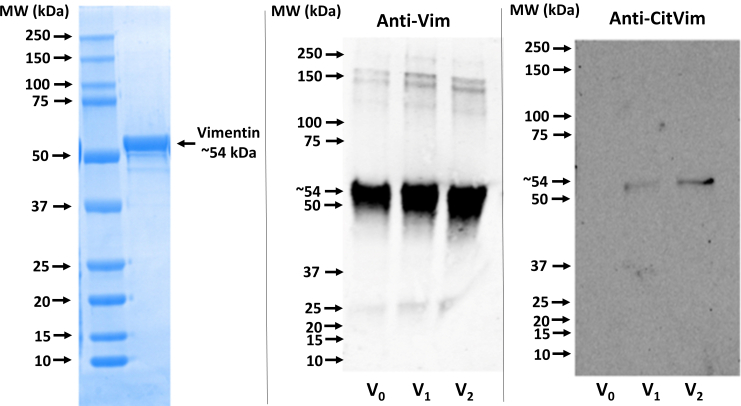
Coomassie-stained SDS-PAGE gel (*left*) and Western blots with anti-vimentin (*middle*) or anti-citrullinated vimentin (*right*). v. VO: vimentin; no PAD4. V1: vimentin + PAD4 low salt; V2 vimentin + PAD4 high salt. PAD4, protein arginine deiminase 4.

To test whether substrate-bound vimentin was sufficient for cell adhesion, we prepared inert polyacrylamide (PAAm) hydrogels, which resist cell adhesion, and covalently laminated their surface with vimentin, fibronectin, or collagen I (Fig. S1). Fig. S1*A* shows a schematic of the protein-coated gel substrate. The purity of the bacterially expressed vimentin is shown in Fig. S2. Previous studies have shown that surface treatment of these gels with an integrin ligand or other cell attachment factor is necessary for cells to form stable adhesions and survive on these surfaces (30). To confirm binding of vimentin to the PAAm gel surface, we stained either collagen-coated (Fig. S1*B*) or vimentin-coated (Fig. S1*C*) PAAm gels with anti-vimentin antibodies and detected vimentin only on the surface of the vimentin-coated gel with immunofluorescence imaging. The surface coating of these gels is relatively uniform but also shows some submicron scale texture due to the polymeric state of the vimentin and the imperfect reaction of the linker group with the PAAm gel surface. These gels were also used to detect possible binding of soluble vimentin to a collagen-coated substrate. The term soluble vimentin denotes nonfilamentous or network forming vimentin, which can exist in solution as oligomers of various sizes and structures, depending on the exact solution conditions. Rhodamine-labeled vimentin does not bind directly to an uncoated PAAm gel (Fig. S1*D*), but it binds to gels that are covalently laminated with collagen I (Fig. S1*E*).

**Figure 1:**
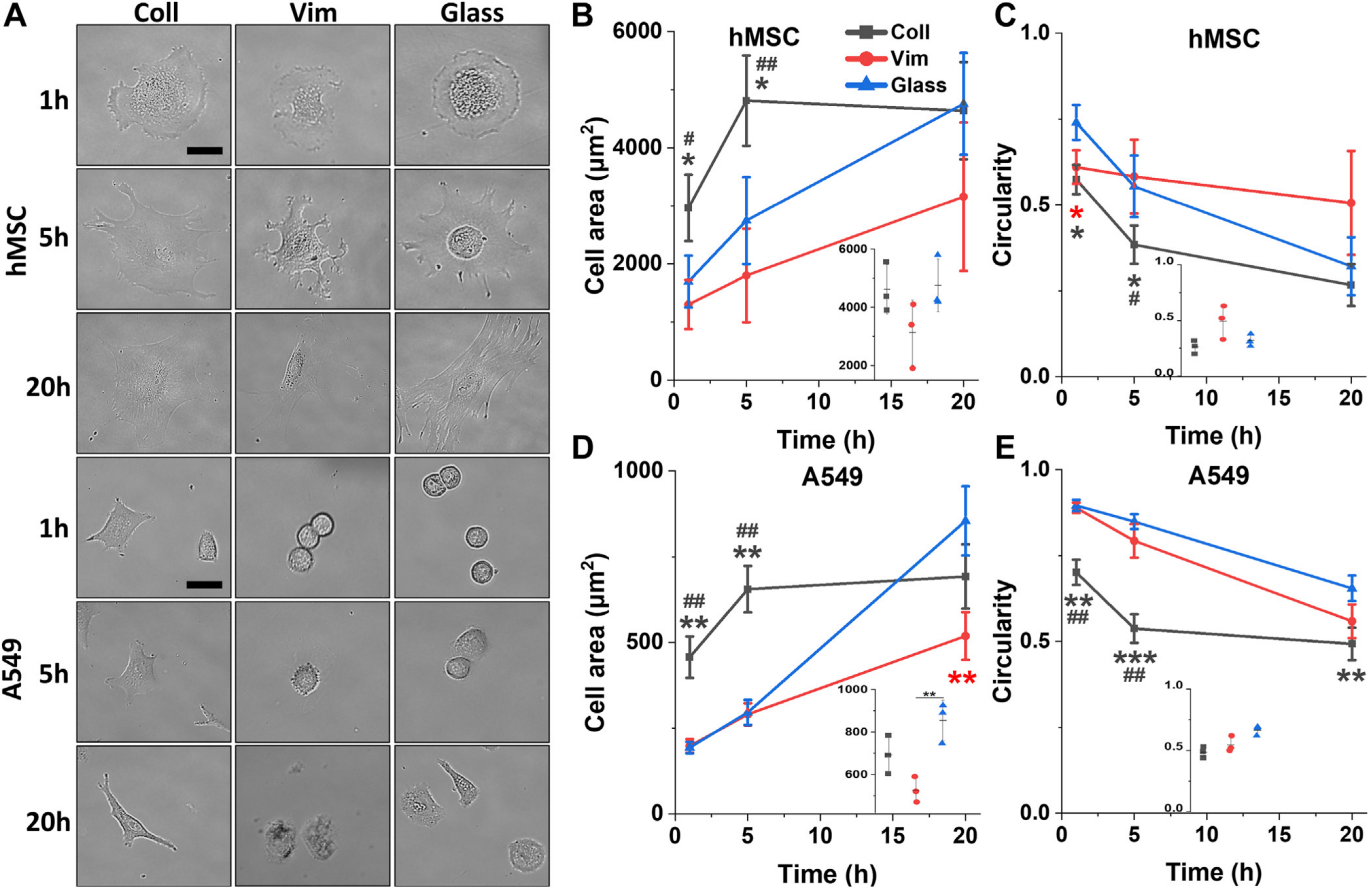
**Morphology and kinetics of spreading of hMSCs and A549 cells on glass or 30 kPa PAAm gels coated with collagen or vimentin.***A*, live cell images of normal or vimentin-null mEFs after 20 h on 30 kPa vimentin-coated PAAm gels. *B*–*E*, time course of changes in spread area and circularity of hMSCs and A549 cells on glass or 30 kPa PAAm gels coated with collagen or vimentin. *Dot plots* incorporated within each figure show data distribution of the corresponding graph at 20 h. Data present mean ± SD from three independent experiments (≥30 and ≥40 cells/experiment for hMSC and A549, respectively). *Asterisks* denote statistical significance compared to cells cultured on glass, and the *hash* sign indicates a comparison between cells on collagen and vimentin. ∗, #*p* < 0.05, ∗∗, ##*p* < 0.01, ∗∗∗*p* < 0.001, by one-way ANOVA with Tukey’s test. Scale bar, 20 μm. hMSCs, human mesenchymal stem cells; mEF, mouse embryonic fibroblast; PAAm, polyacrylamide.

Figure 1 shows that human mesenchymal stem cells (hMSCs) and A549 lung cancer cells adhere and spread on vimentin-coated PAAm gels. Both the kinetics and extent of cell spreading on vimentin-coated gels are different from the spreading rate on collagen-coated gels or glass, suggesting that the receptors and downstream signals activated by engagement of vimentin might be distinct from those that govern cell adhesion to integrin ligands.

**Figure 2:**
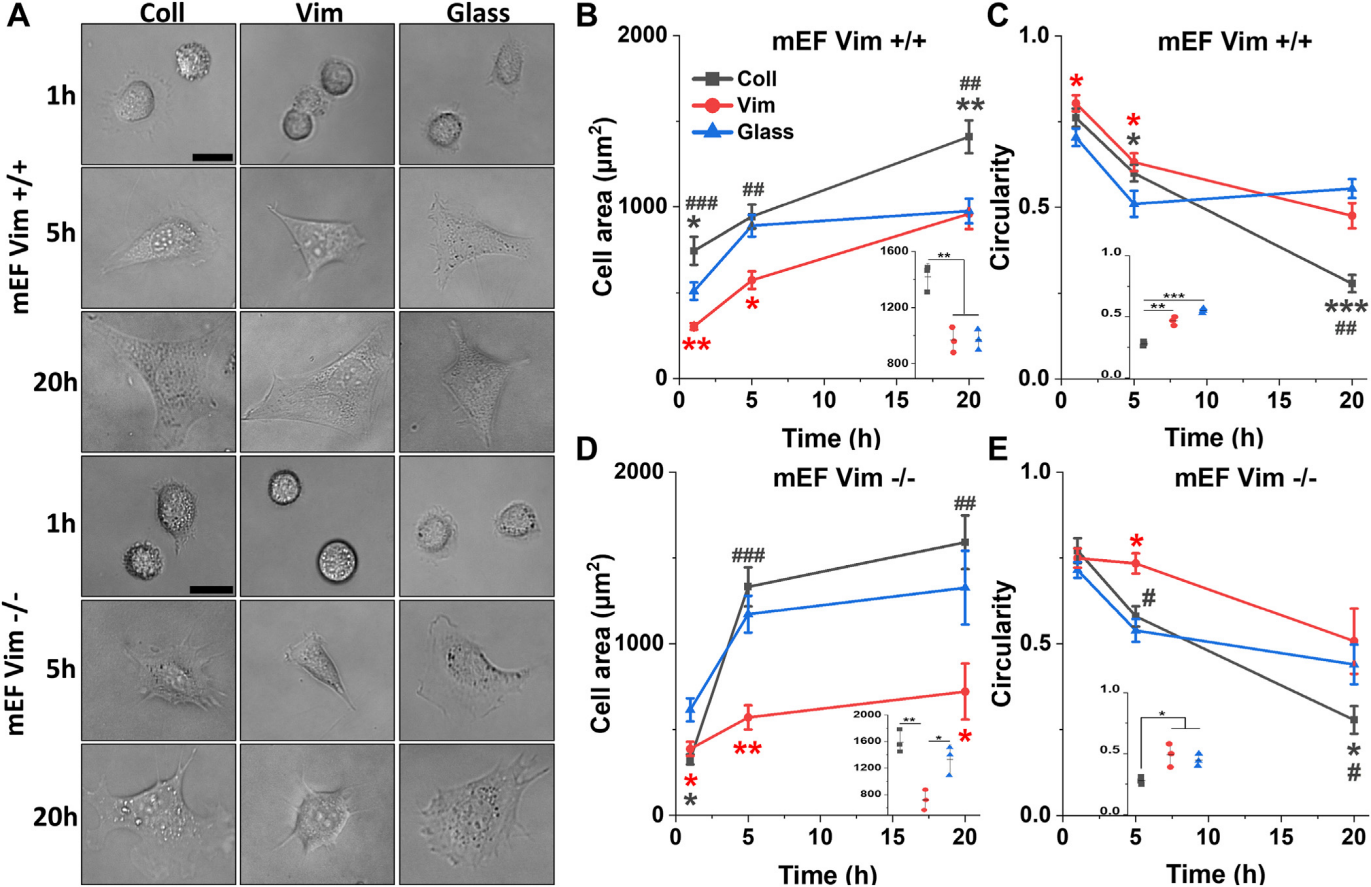
**Morphology and kinetics of spreading of vim +/+ and vim −/− mEFs on glass or 30 kPa PAAm gels coated with collagen or vimentin.***A*, live cell images of normal or vimentin null mEFs after 20 h on 30 kPa vimentin-coated PAAm gels. *B*–*E*, time course of changes in spread area and circularity of vim +/+ and vim −/− mEFs on glass or 30 kPa PAAm gels coated with collagen or vimentin. *Dot plots* incorporated within each figure show data distribution of the corresponding graph at 20 h. Data presents mean ± SD from three independent experiments (≥65 and ≥50 cells/experiment for mEF vim +/+ and mEF vim −/−, respectively). *Asterisks* denote statistical significance compared to cells cultured on the glass, and the *hash* sign indicates a comparison between cells on collagen and vimentin. ∗, #*p* < 0.05, ∗∗, ##*p* < 0.01, ∗∗∗, ###*p* < 0.001, by one-way ANOVA with Tukey’s test. Scale bar, 20 μm. mEF, mouse embryonic fibroblast; PAAm, polyacrylamide.

**Supporting Figure S3:**
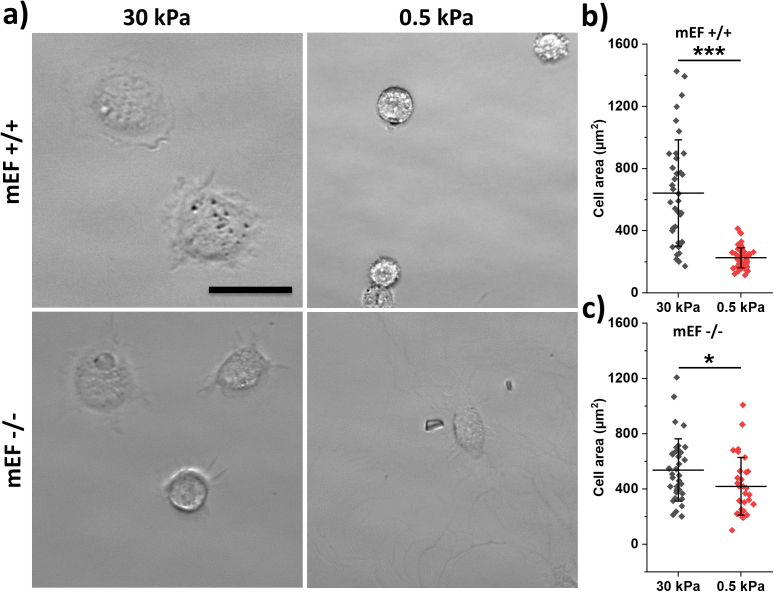
Morphology (*A*) and cell area of mEF +/+ (*B*) and mEF −/− (*C*) cells after 1 h incubation on a 0.5 kPa and 30 kPa PAAm gels coated with vimentin. Data present mean ± SD (≥30 cells/experiment). ∗*p* ≤ 0.05, ∗∗∗*p* < 0.001, by Student’s *t* test. Scale bar, 20 μm. mEF, mouse embryonic fibroblast; PAAm, polyacrylamide.

Figure 2 shows that both normal and vimentin null mouse embryonic fibroblasts (mEFs) adhere to vimentin-coated 30 kPa PAAm gels and spread on them to form flattened cell structures, similar in size to those they would form on integrin ligand–coated gels or glass or plastic surfaces. The similar morphologies of normal and vimentin-null fibroblasts on vimentin-coated substrates show that adhesion of these cells to substrate-bound vimentin does not require vimentin–vimentin contacts, which might occur in vimentin-positive cells that also express vimentin on their surface. Therefore, the vimentin adhesion receptor on these cells appears to be distinct from vimentin itself and might function either similarly or differently from other cell adhesion proteins, such as integrins. Fibroblasts bound to vimentin-coated gels are capable of mechanosensing, since the spread area of cells on 0.5 kPa vimentin-coated gels was much smaller than those on 30 kPa gels (Fig. S3). Lack of integrin activation by vimentin-coated gels is also supported by the observation that no cell division was seen over 20 h on vimentin-coated gels, whereas 5% of the fibroblasts divided on collagen- or fibronectin-coated gels.

### Cytoskeletal changes and adhesion sites in vimentin substrate–bound cells

**Figure 3:**
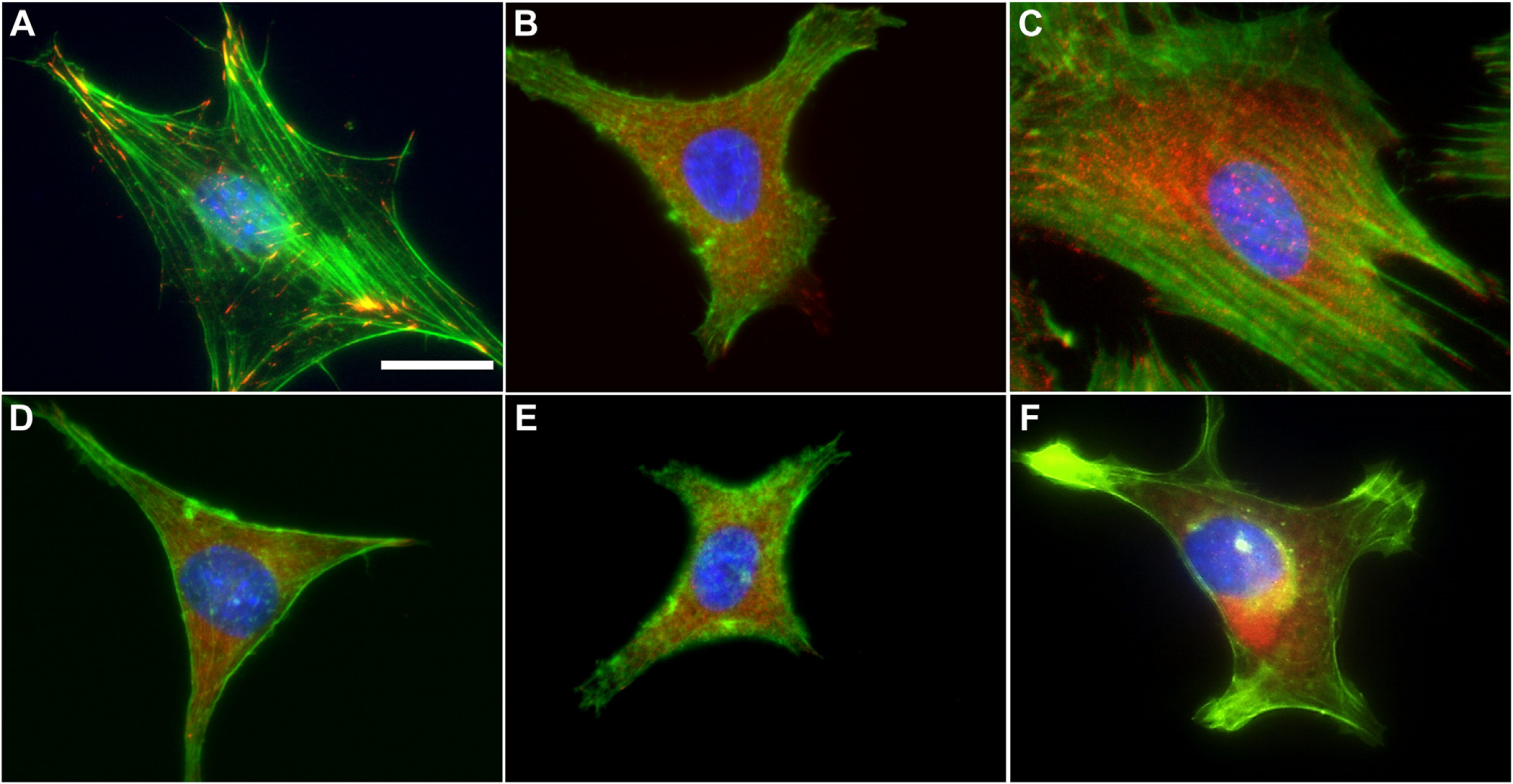
**Fluorescence images.***A*–*F*, mEF Vim +/+ (*A* and *D*), A549 (*B* and *E*), and hMSC (*C* and *F*) cells seeded on 30 kPa hydrogels coated with collagen (*A*–*C*) or vimentin (*D*–*F*). Vinculin is presented in *red*, F-actin in *green*. Cells were counterstained with DAPI (*blue*). Scale bar, 20 μm. hMSC, human mesenchymal stem cell; mEF, mouse embryonic fibroblast.

Consistent with the hypothesis that cell adhesion to vimentin-coated surfaces is distinct from adhesion to collagen or other integrin ligands, Figure 3 shows that although cells spread on both collagen-coated and vimentin-coated surfaces, the structures of their actin and vimentin filament networks are different. The adhesion sites that these cells form are also different. Figure 3, *A*–*C* show that the formation of large actin bundles, or stress fibers, is a common feature of fibroblasts and MSCs binding to stiff collagen-coated gels, but these structures do not appear, or at least are much less developed, in cells spread to similar areas on vimentin-coated gels (Fig. 3, *D*–*F*). Vinculin is more localized to actin-containing fibers and less diffuse for cells on collagen (A–C) compared to cells on vimentin (D–F). A549 lung cancer cells appear to be different. They form actin bundles on both collagen-coated (C) and vimentin-coated gels (E), although the vinculin staining is less diffuse on collagen-coated gels than vimentin-coated ones (E).

### Traction stresses on vimentin-coated deformable substrates

**Figure 4:**
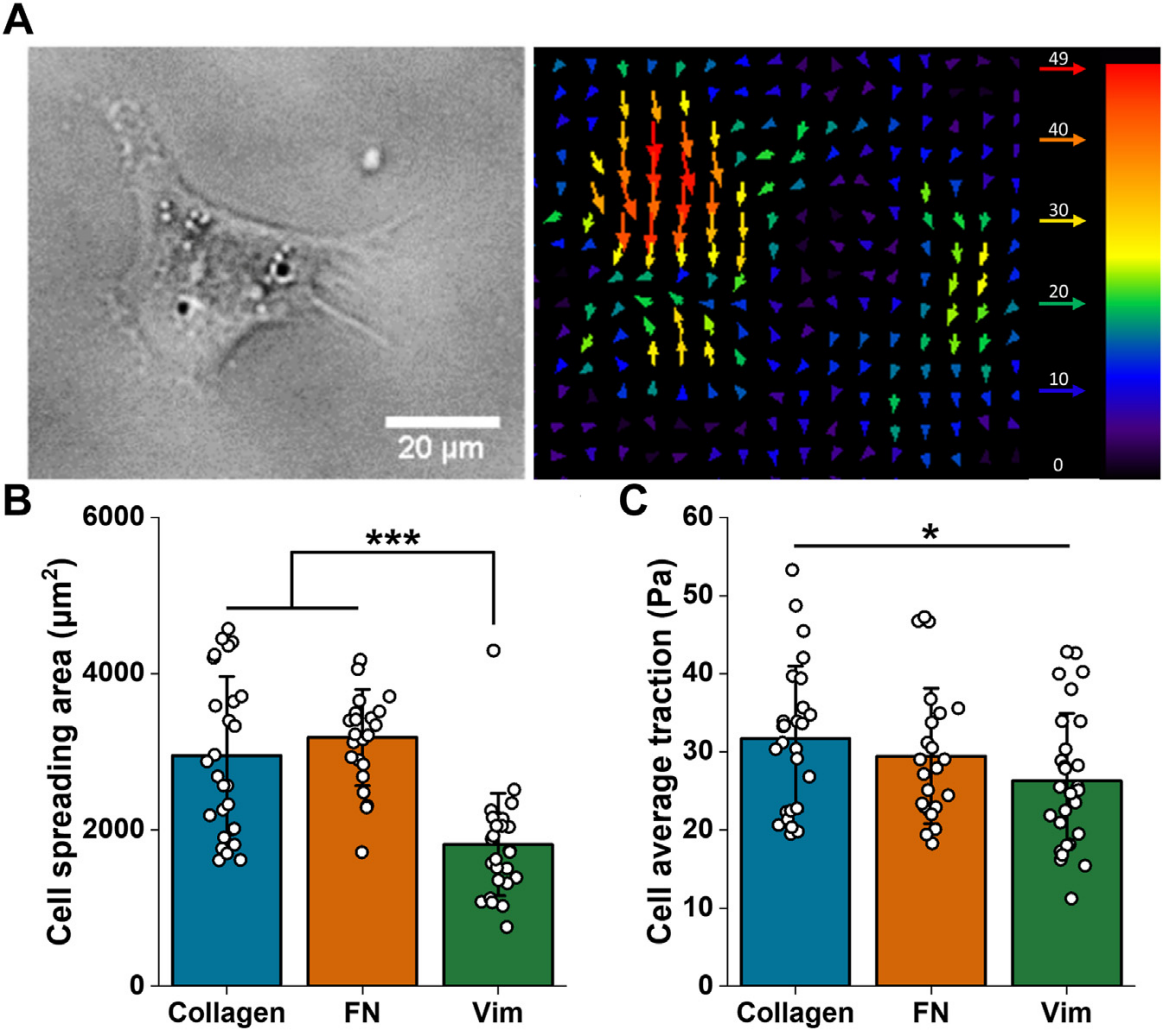
**Adhering hMSCs on 30 kPa vimentin-coated PAAm gel exhibit traction forces.***A*, bright-field image and traction force map of a representative hMSC cultured on a vimentin-coated gel. *B* and *C*, average cell spreading area (*B*) and average traction force (*C*) of hMSCs cultured on gels coated with collagen, fibronectin, or vimentin. Data present mean ± SD, N > 22. ∗*p* < 0.05, ∗∗∗*p* < 0.001 by one-way ANOVA with Tukey’s test. hMSC, human mesenchymal stem cell; PAAm, polyacrylamide.

Despite the lack of actin stress fibers or large focal adhesions in cells bound to vimentin-coated gels, these cells nevertheless exert traction stresses on the substrate, consistent with their ability to mechanosense (Fig. S3). Fig. 4*A* shows a typical MSC bound to a vimentin-coated gel and a typical strain field generated by the cells on the deformable substrate using traction force microscopy. Figure 4*B* shows the average spread area on gels coated with collagen, fibronectin, or vimentin, and Figure 4*C* shows that the magnitude of traction stress that cells exert on vimentin-coated surfaces is significant but smaller than the traction stresses on collagen- or fibronectin-coated substrates. These results are consistent with the morphology shown in Figure 1, Figure 2, Figure 3 and confirm that the nature of the adhesion of cells to vimentin-coated substrates is different from that of integrin-dependent adhesion.

### Extracellular vimentin promotes cell migration through ECM networks

**Figure 5:**
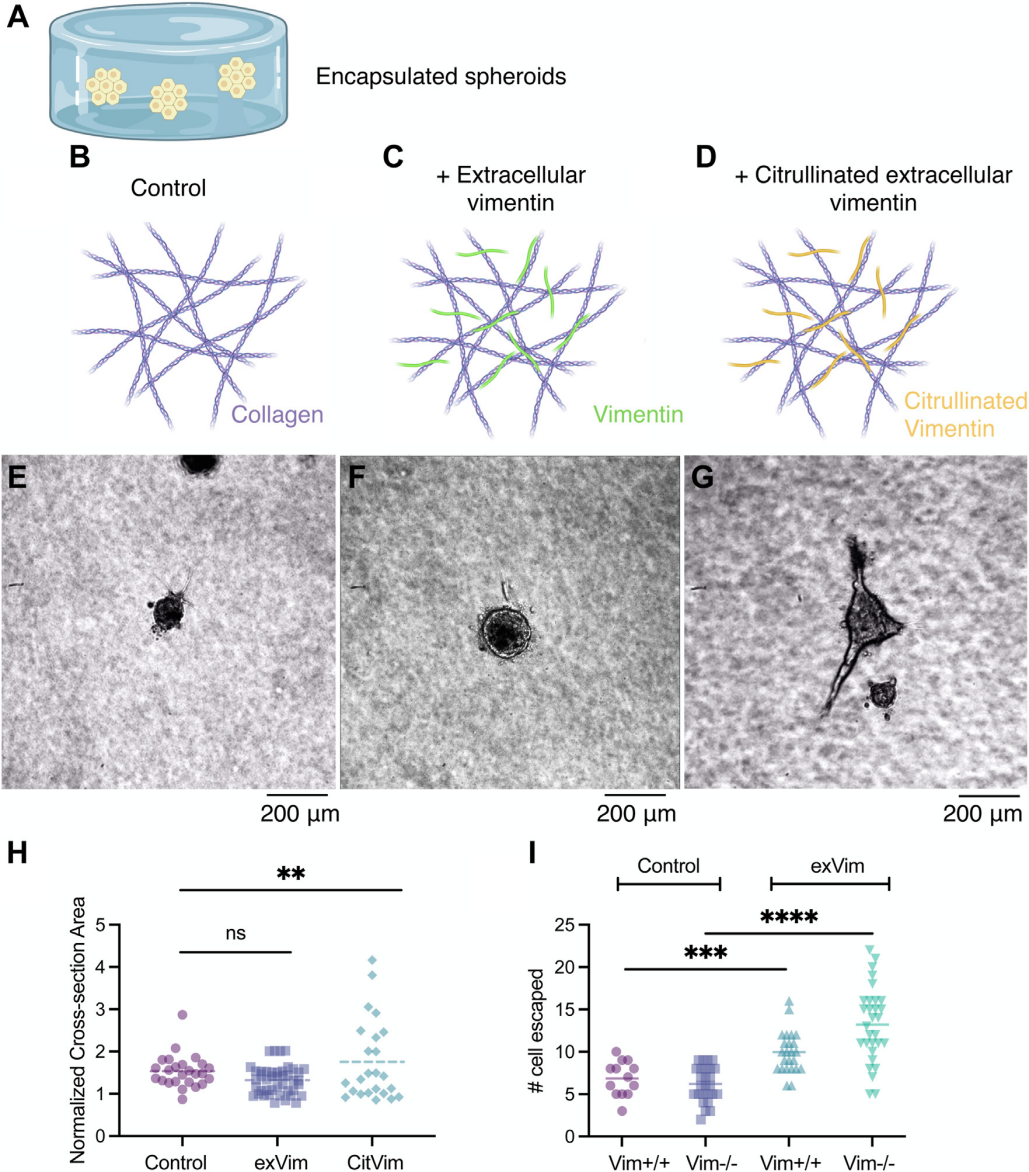
**Extracellular vimentin promotes cell migration through ECM networks.***A*, experimental method. Cellular aggregates are embedded in collagen I gels (2 mg/ml). Cartoon representing the matrix presentations to the cells: *B*–*D*, the control collagen I matrix (*B*), collagen I with extracellular vimentin (2 ug/ml) (*C*), and collagen I (*D*) with extracellular vimentin in its citrullinated form (2 ug/ml). *E*–*G*, representative phase-contrast images of cell aggregates formed by wildtype mEF embedded in matrixes containing (*E*) collagen I, (*F*) collagen I + vimentin, and (*G*) collagen I + citrullinated vimentin at 24 h. *H*, averaged projected area of cell aggregates formed from wildtype mEF embedded in collagen network at 42 h (N ≥ 3 trials, n ≥ 23 spheroids; Statistical test: one way ANOVA: ns: *p* = 0.2602, ∗∗*p* = 0.008). *I*, single cell escape data for wildtype and vimentin-null MEF aggregates for each condition (collagen, collagen+vimentin, collagen+citrullinated vimentin). Data collected at 48 h. Addition of extracellular vimentin to the network increases number of cells escaping from aggregates for both cell types (Stat test Mann-Whitney U: ∗∗∗*p* = 0.0004; ∗∗∗∗*p* ≤ 0.0001). ECM, extracellular matrix; mEF, mouse embryonic fibroblast.

Vimentin released to the extracellular space is at least partly posttranslationally modified, often by phosphorylation or citrullination (reviewed in (11)), and these covalently modified forms of vimentin appear to function differently from unmodified vimentin (31). Previous studies suggest that citrullinated vimentin is more effective than unmodified vimentin in promoting motility of cells through extracellular matrices (31). To determine whether extracellular vimentin mediates cell motility through three-dimensional (3D) fibrillar collagen matrices, we prepared spheroidal aggregates of mEFs embedded in 2 mg/ml collagen I networks with the addition of either vimentin or its citrullinated form (2 ug/ml) to the collagen network (Fig. 5). Vimentin binds collagen as indicated by Fig. S1, and immunofluorescence staining showed vimentin was incorporated into the collagen network. We found that the addition of extracellular vimentin (in contrast to its citrullinated form) did not change the average spheroidal aggregate area over time but did impact how cells migrated into the collagen gel, which was individually rather than as strands of connected cells (Fig. 5, H and I). Using time-lapse imaging of the embedded cell aggregates, we observed that invading strands of cells form in the presence of extracellular vimentin, but many of them disassemble over the course of 24 h, similar to the effect of vimentin on endothelial cells (19). In contrast to normal vimentin in the network, citrullinated vimentin significantly increased collective invasion of wildtype mEFs into the collagen network, as shown in Figure 5. This is consistent with the effect of citrullinated vimentin on primary human lung fibroblast migration (31), and here we note markedly large strands of cells that emerge from the aggregate in the presence of the additional citrullinated vimentin in the network (Fig. S4). The invasion of spheroidal aggregates formed of vimentin-null mEFs into the collagen network is similar to that of aggregates formed of wildtype mEFs. Addition of unmodified vimentin and citrullinated vimentin to the collagen network did not increase vimentin-null aggregate spread areas but did increase the number of individual cells that escape into the network.

### Effects of glycocalyx and GlcNAc on adhesion to vimentin-coated substrates

**Figure 6:**
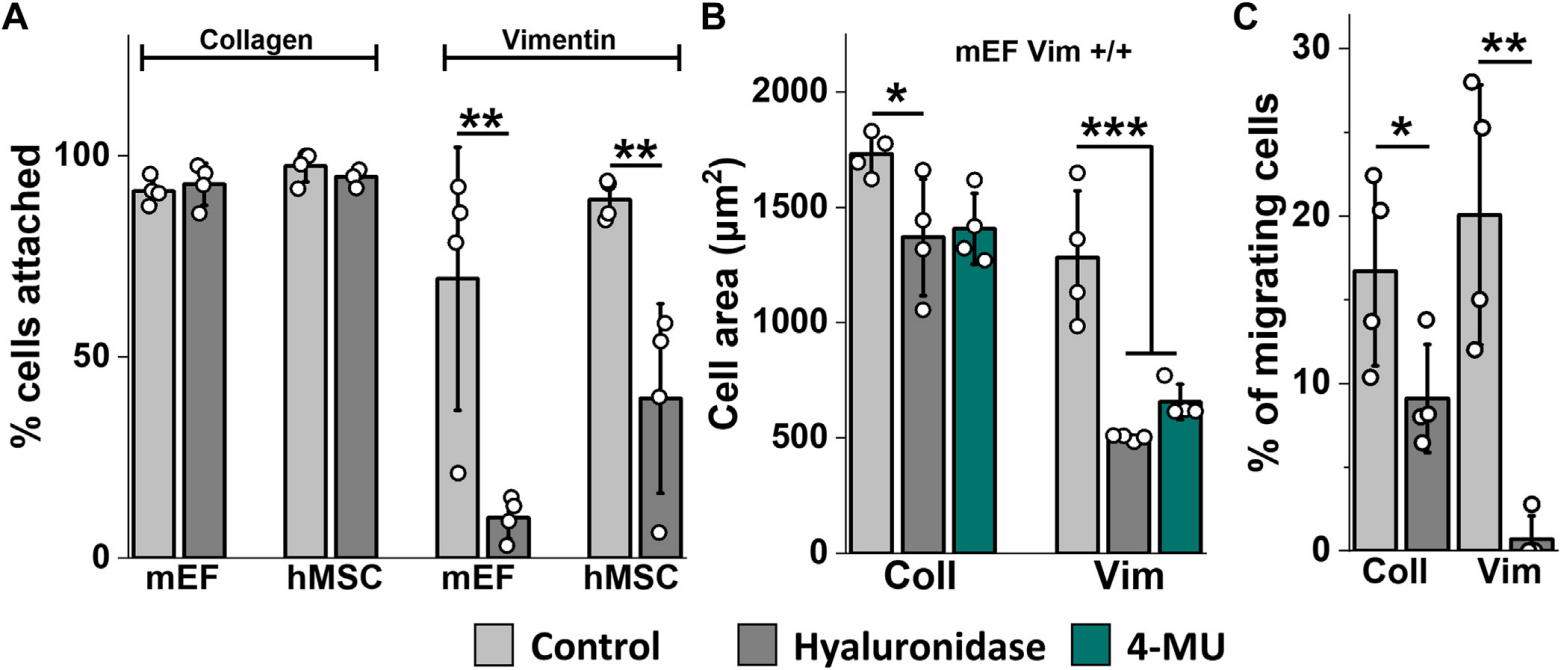
**Cell attachment, cell area, and cell migration on vimentin-coated substrates.***A*, fibroblast and hMSC attachment is inhibited by hyaluronidase on vimentin-coated but not collagen-coated PAA gels. *B*, average cell area of mEFs cultured on PAA gels coated with collagen (*left*) or vimentin (*right*) ± hyaluronidase (10U/mL) or 4-methylumbelliferone (4-MU) (3 mM). *C*, hMSC migration on vimentin-coated gels is inhibited by hyaluronidase more than on collagen-coated gels. Data present mean ± SD from four independent experiments (five images at 10× magnification (*A* and *C*), ≥30 cells/experiment (*B*). ∗*p* < 0.05, ∗∗*p* < 0.01, ∗∗∗*p* < 0.001, by (*A* and *C*) Student’s *t* test or (*B*) one-way ANOVA with Tukey’s test. hMSC, human mesenchymal stem cell; mEF, mouse embryonic fibroblast.

Based on previous reports that vimentin can bind HA (32) and heparin (33), constituents of the cells’ glycocalyx, we tested the effect of hyaluronidase, which degrades the glycocalyx, and the drug 4-methylumbelliferone, which inhibits synthesis of the glycocalyx by the membrane bound enzyme HA synthase (34) and blocks the ability of cultured endothelial cells to respond to fluid shear stress (35). Figure 6*A* shows that hyaluronidase treatment strongly decreases the adhesion of fibroblasts and MSCs to vimentin-coated substrates but has no effect on the adhesion of the same cell types to collagen-coated gels. Figure 6*B* shows that cells that do attach to vimentin-coated gels after inhibition of glycocalyx production have significantly smaller spread area, but the effect is not statistically significant for cells on collagen-coated gels. In addition, we tested the effect of hyaluronidase treatment on the motility of cells on collagen- or vimentin-coated substrates. Figure 6*C* shows that the motility of hMSCs was almost completely blocked by hyaluronidase, whereas there was a much smaller effect on the motility of cells on collagen-coated cells, an effect that is expected because of the interplay between the glycocalyx and integrins (36).

**Figure 7:**
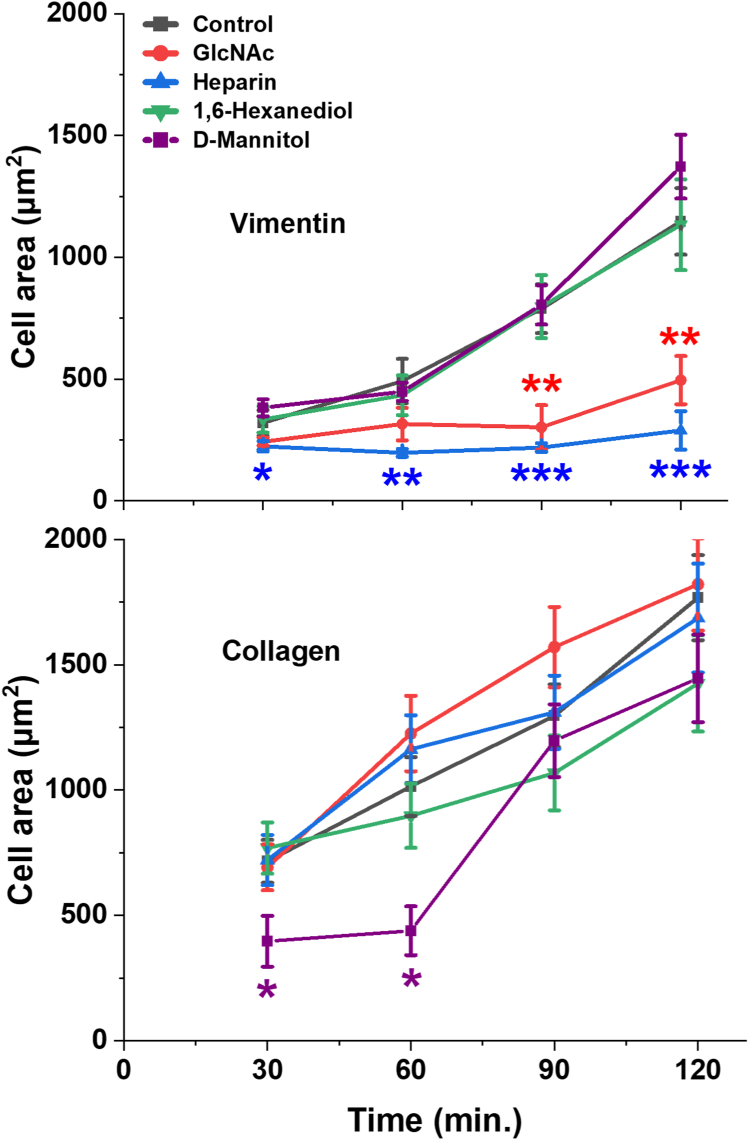
**Effects of the glycocalyx on cell spreading dynamics on vimentin-coated substrates.***Top panel*: Fibroblast spreading is inhibited by GlcNAc and heparin, but not 1,6 hexanediol and D-mannitol on vimentin-coated 30 kPa PAAm gels. *Bottom panel*: No effect on cells bound to collagen-coated gels. Data present mean ± SD from three independent experiments (≥40 cells/experiment). ∗*p* < 0.05, ∗∗*p* < 0.01, ∗∗∗*p* < 0.001, by Student’s *t* test. GlcNAc, N-acetyl glucosamine; PAAm, polyacrylamide.

Previous studies have shown that cells are able to bind otherwise nonadhesive surfaces that are coated with polymers containing GlcNAc but not other sugars and that the receptor on the cell surface, which acts like a lectin, is vimentin (37). Cell adhesion in that study was blocked if the cells were treated with soluble GlcNAc before exposure to the GlcNAc polymer-containing substrate. We therefore tested the effect of soluble GlcNAc on cell adhesion to vimentin-coated hydrogels. Figure 7 shows that soluble GlcNAc strongly inhibits the adhesion and spreading of fibroblasts on vimentin-coated surfaces, but it has no effect on the adhesion of the same cell type to collagen-coated gels. The glycosaminoglycan heparin, which contains GlcNAc subunits, also strongly inhibits binding of cells to vimentin-coated gels but has no effect on collagen-coated gel adhesion. In contrast, 1-6 hexane diol, which has been reported to interfere with vimentin assembly at much higher concentration (38), has no effect on adhesion of cells to either vimentin- or collagen-coated substrates, confirming that the effect of soluble GlcNAc is not due to generic hydrogen-bonding interactions that might be disrupted by a chemical such as 1-6 hexane diol. D-mannitol, previously shown not to affect binding of vimentin-coated cells to GlcNAc-coated surfaces (37), also had no effect.

### Effects of extracellular vimentin on cell adhesion

**Figure 8:**
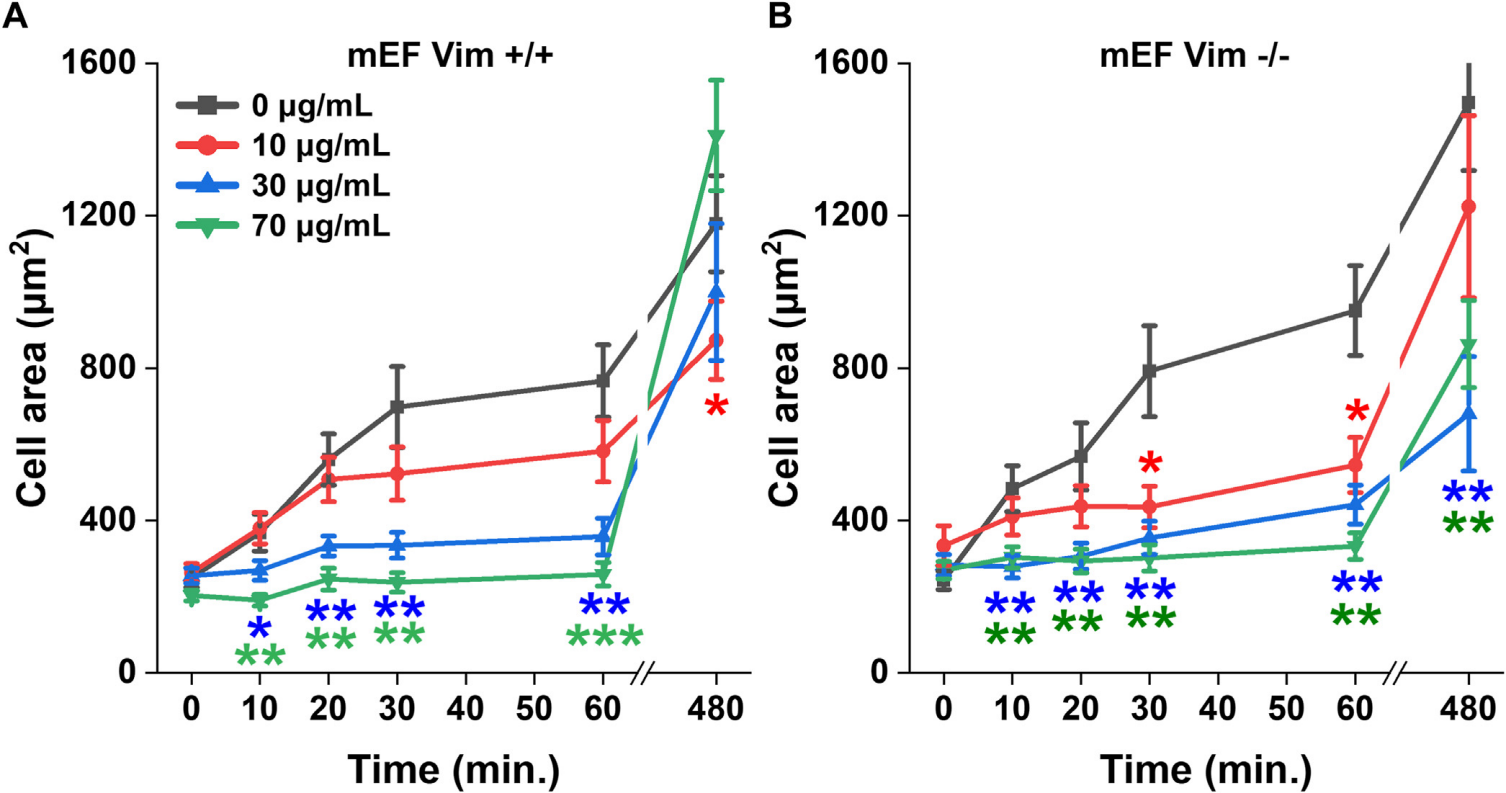
**Effect of soluble vimentin on adhesion and spreading of fibroblasts on glass.***A* and *B*, cell area of (*A*) mEF Vim +/+ and (*B*) mEF Vim −/− cells that were preincubated with vimentin at indicated concentrations. Data present mean ± SD from three independent experiments (≥40 cells/experiment). ∗*p* < 0.05, ∗∗*p* < 0.01, ∗∗∗*p* < 0.001, by Student’s *t* test. mEF, mouse embryonic fibroblast.

Numerous studies have shown that cells release vimentin into the extracellular space in multiple contexts: on the extracellular surface of the cells, attached to the underlying basement membrane, or soluble in the bloodstream or cell culture medium (9, 11, 13, 14). We therefore tested if soluble vimentin had an effect on cell adhesion to substrates that do not already contain vimentin. Figure 8 shows the spreading of fibroblasts on glass when increasing concentrations of vimentin are added in soluble form to the cell culture medium. Both normal and vimentin-null fibroblasts are inhibited from spreading on glass by 10 μg/ml concentrations of soluble vimentin, similar to the levels (∼1 μg/ml) in serum of patients with cancer or sepsis (39, 40). The effect of soluble vimentin appears to be greater on vimentin-null fibroblasts compared to normal fibroblasts, suggesting that perhaps the normal fibroblasts already contain some cell surface–bound vimentin, but since the vimentin-null fibroblasts do not, they are affected by smaller doses of exogenously added vimentin.

## Discussion

Extracellular vimentin is expressed in several pathophysiological conditions, and several reports indicate its role in mediating response to cellular damage or as an autoantigen in rheumatoid arthritis (17, 41). Studies to identify the cellular receptor for extracellular vimentin have proposed numerous candidate protein ligands. Cell surface vimentin has been implicated in platelet recruitment at injury sites *via* interaction with von Willebrand Factor. Disruption of this interaction, either with anti-vimentin antibodies or with a von Willebrand Factor fragment, positively affected experimental models of ischemic stroke (42). Other reports indicate vimentin interaction with P-selectin, dectin-1, CD44 (43) and insulin-like growth factor 1 receptor (44, 45, 46). Numerous pathogens, including bacteria and viruses, express distinct vimentin-binding proteins on their surface, and these facilitate the binding and entry of the pathogen to the eukaryotic host (reviewed in (11, 13, 47)). The variety of proteins reported to mediate binding of extracellular vimentin to the cell surface suggests that these proteins might act together with some other common membrane structure, such as the glycocalyx, or that these proteins share some common feature such as glycosylation. Previous reports that vimentin binds GlcNAc-containing macromolecules (37) and the importance of O-linked GlcNAc on the structure of the vimentin cytoskeletal network (48, 49, 50, 51) suggest that these or related glycosylated structures might mediate cell binding to extracellular vimentin.

The appearance of vimentin in the extracellular space occurs during early development, wound healing, inflammation, and some types of cancer. For example, binding of extracellular vimentin to dectin-1, a marker of activated macrophages, triggers reactive oxygen species production (46, 52). Extracellular vimentin amplifies the secretion of proinflammatory cytokines, including IL-6 and TNF-α, induced by oxLDL in macrophages (20). In contrast, LPS-activated dendritic cells, when exposed to vimentin, switch their cytokine profile, decreasing IL-6 and IL-12 secretion, while increasing anti-inflammatory IL-10, thereby reducing Th1 differentiation of naïve T cells (53). The nonessential aspect of extracellular vimentin is consistent with the finding that vimentin-null mice develop nearly normally (54) and are relatively resistant to viral or bacterial infection or to catastrophic inflammatory responses to endotoxin (55), two conditions in which extracellular vimentin is normally released onto the cell surface or into the extracellular space (12).

In this study, we show that vimentin on an otherwise nonadhesive hydrogel is sufficient for the adhesion of multiple different cell types to the substrate. A limitation to the present data is that the cell types studied have a mesenchymal origin, and even vimentin-null fibroblasts might possess a vimentin-binding partner that nonmesenchymal cell types that only express other IF classes lack. Binding of cells to vimentin-coated substrates appears to involve different receptors from those used to bind collagen or fibronectin because it is inhibited or eliminated either by removal of the cells’ HA-containing glycocalyx or by addition of soluble GlcNAc to the substrate, two treatments that have little or no effect on cell binding to collagen- or fibronectin-coated surfaces. The effect of GlcNAc on extracellular vimentin might be structurally related to the role of O-linked glycosylation of specific residues such as Ser34, 39, and 49 by GlcNAc on cytoskeletal vimentin. These sites localize to the unstructured N terminus, where phosphorylation of some of the same serines leads to vimentin filament disassembly. In contrast, glycosylation at Ser 49 promotes binding of the N-terminal head to an adjacent alpha helix in another subunit of the filament (49, 51). Other IF proteins are also modified by GlcNAc at equivalent serines. These modifications promote interactions with other cellular proteins, for example enhancing interaction of desmin with αB-crystallin (56) or keratin 8 with AKT (50, 57). The vimentin used in the present study was bacterially expressed and therefore presumably devoid of glycosylation, and a possible effect of endogenous glycosylation in the function of extracellular vimentin has not yet been reported.

Once bound to vimentin-coated substrates, cells can spread and move, but not divide. Most cell types tested spread more slowly on vimentin than they do on collagen-coated surfaces, but in some cases, they move more rapidly. The effect of vimentin on motility is also evident when cell spheroids are placed within three-dimensional collagen networks. The addition of exogenous vimentin, and especially its citrullinated form, causes cells to leave the spheroid and stream out into the collagen network much more efficiently than they would in the absence of exogenous vimentin. These findings confirm the previous reports that extracellular vimentin is an important driver of the movement of fibroblasts into inflamed lung tissue.

In summary, our data show that extracellular vimentin is sufficient to support cell adhesion and motility, and cells bound to vimentin on a hydrogel substrate exert traction stresses and are capable of mechanosensing. The morphology and motility of cells bound to vimentin-coated surfaces is different from that of cells bound to the same substrates coupled only to collagen. Addition of vimentin, especially citrullinated vimentin, to three-dimensional collagen networks strongly enhances the migration of cells out of spheroidal aggregates into the matrix, suggesting that different adhesion receptors engage vimentin and collagen. Disruption of the cell surface glycocalyx or addition of soluble GlcNAc to the cell culture medium disrupts cell binding to vimentin-coated surfaces but does not disrupt binding to collagen. These results reveal a novel aspect of the interaction of cells with their ECM, especially in inflammatory states where vimentin is disassembled and released into the extracellular space.

## Experimental procedures

### Reagents

D-glucuronic acid (G526), 4-methylumbelliferone (M1381), HA sodium salt from *Streptococcus equi* mol wt 15,000 to 30,000 (97616), 1,6-Hexanediol (H11807), N-acetyl-D-glucosamine (A3286), heparin sodium salt from porcine intestinal mucosa (H3393), D-mannitol (M4125), (3-aminopropyl)trimethoxysilane (281778), and 70% (v/v) glutaraldehyde (G7776) were from Sigma-Aldrich. 2% (w/v) bis acrylamide (1610142) and 40% (w/v) acrylamide solutions (1610140) were from Bio-Rad Laboratories. TEMED (TB0508) was from Bio Basic. Ammonium persulfate (17-1311-01) was from Life Sciences, and hyaluronidase from *Streptomyces hyaluronlyticus* (389561) was from Millipore Corp. Collagen I (354236) was from BD Bioscience. Sulfo-SANPAH crosslinker (A35395) was from Thermo Fisher Scientific. Recombinant human protein arginine deiminase 4 (PAD4) was from Cayman Chemical, Inc.

### Preparation of vimentin

Vimentin-coding DNA was cloned into the pET7 plasmid (provided by Robert Goldman, Northwestern University), transformed into *Escherichia coli* BL21(DE3) competent cells, and grown on LB-Agar plates containing 100 mg/ml ampicillin. Single colonies from the LB-Agar plates were inoculated into LB medium containing 100 mg/ml ampicillin and grown overnight at 37 °C. The overnight cultures were used to inoculate a 10 l bioreactor containing LB medium at 37 °C that was agitated at 600 RPM. Protein production was induced by the addition of IPTG to a final concentration of 100 mM, and the cultures were left overnight in the bioreactor. Cells were harvested and resuspended in lysis buffer (50 mM Tris-HCl (pH 8.5), 0.5 M NaCl, benzonase and lysozyme). The resuspended cells were lysed by sonication, and the suspension was centrifuged on a JS-4.2 rotor (Beckman) at 6000 RPM for 30 min. The pellet was washed successively in wash buffer-1 [50 mM Tris-HCl (pH 8.5), 1 M NaCl, 0.1% (v/v) Triton X-100, 2 M urea] and wash buffer-2 [50 mM Tris-HCl (pH 8.5), 1 M NaCl, 2 M urea)] by resuspension and centrifugation on a JS-4.2 rotor (Beckman) at 6000 RPM for 30 min. The washed pellet was resuspended in solubilization buffer [50 mM Tris-HCl (pH 8.5), 8 M urea] by stirring overnight at room temperature. The solubilized pellet was loaded on a HiTrap Q FF column (5 ml volume; Cytiva) that had been equilibrated in solubilization buffer and eluted in the same buffer using a 0 to 1 M gradient of NaCl. Fractions containing vimentin were pooled, dialyzed against 50 mM Tris pH 8.5 + 6 M guanidine HCl, and snap-frozen in liquid nitrogen. The purity of vimentin was assessed using SDS-PAGE (Fig. S1). A fraction of the vimentin preparation was labeled with rhodamine B-succinimide by the method previously used to label neurofilaments (58).

### Citrullination of vimentin

The citrullination procedure was based on a method for citrullinating fibrinogen (59). Purified vimentin (4 mg/ml) was dialyzed into 10 mM Tris-HCl, 1 mM EDTA, and 6 mM DTT, pH 7.6, a low ionic strength buffer that destabilizes vimentin polymerization (V1). A portion of vimentin was supplemented with 2 mM MgCl_2_ and 150 mM KCl to polymerize it (V2). Lyophilized PAD4 was suspended in 40 mM Tris-HCl, 5 mM CaCl_2_, and 10 mM DTT, pH 7.5, to a concentration of 3 mg/ml. Vimentin and PAD4 were mixed to concentrations of 3.8 mg/ml and 0.13 mg/ml, respectively, and incubated 12 h at 37 °C. Purity of the vimentin preparations and evidence of its citrullination are shown in Fig. S1. As reported previously (60), vimentin citrullination results in filament disassembly under polymerizing conditions.

### Preparation of vimentin-, collagen-, and fibronectin-coated substrates

Cells were cultured on PAAm hydrogel substrates of 0.5 and 30 kPa stiffness that were prepared using the methods previously described (61, 62). The acrylamide and bis-acrylamide solutions were formulated in distilled H_2_O to a total volume of 1 ml. 1 μl of TEMED and 3 μl of 2% (w/v) ammonium persulfate were used as polymerization initiators. The solution (30 μl) was deposited on a 12-mm square glass coverslip pretreated with 3-aminopropyltrimethoxysilane and 0.5% (v/v) glutaraldehyde. After removing the top coverslip, PAAm gels were covalently linked to ligands by incubating the gels with 50 μl of 0.1 mg/ml collagen I, 0.1 mg/ml fibronectin, or 0.1 mg/ml vimentin after activating the gel surface with the UV-sensitive Sulfo-SANPAH crosslinker. All three proteins were dialyzed against PBS and diluted to 0.1 mg/ml from solutions with concentrations of 3 mg/ml (vimentin), 10 mg/ml (collagen), and 2 mg/ml (fibronectin) into a much larger volume of 50 mM Hepes, pH 8.5. As a result, the solution of vimentin overlaid on the activated gel surface contained 47.6 mM Hepes, pH 8.5, 6.5 mM NaCl, 0.47 mM phosphate, and 0.12 mM KCl. The solutes for the other proteins were essentially the same. The stiffness of the gel substrates was verified using atomic force microscopy (data not shown).

### Cell types and cell culture

Wildtype and vimentin-null mouse embryonic fibroblasts provided by J. Eriksson (Abo Akademi University) were maintained in Dulbecco’s Modified Eagle’s Medium (DMEM) + 4.5 g/l glucose + 2 mM L-glutamine + sodium pyruvate (Corning), supplemented with 10% (v/v) fetal bovine serum (Hyclone), 1% (w/v) nonessential amino acid (Fisher Scientific), 25 mM Hepes (Fisher Scientific), 100 μg/ml streptomycin, and 100 units/ml penicillin (Fisher Scientific). Cell cultures were maintained at 37 °C with 5% CO_2_. Cells were passaged every 3 to 4 days and harvested for experiments when 70% confluent. The lung carcinoma cell line A549 CCL-185 (ATCC) was cultured in DMEM (Gibco) supplemented with 10% fetal bovine serum (FBS, Gibco), 100 U/ml penicillin, and 100 μM streptomycin (Sigma-Aldrich) on tissue culture plastic and kept in a humidified incubator at 37 °C and 5% CO2. hMSCs (Lonza) were cultured in the same conditions in their respective medium for a period of 24 h or greater.

### Adhesion, motility, and proliferation studies by live cell imaging

Cell area, circularity, and proliferation were determined from optical images collected with 10x or 40x air lenses and phase contrast using a Hamamatsu camera on a Leica DMIRE2 inverted microscope (Leica). For each condition, a minimum of 5 (10×) to 20 (40×) images were acquired, and approximately 100 to 150 cells per condition were analyzed. Cell area was calculated using Fiji software by tracing cell peripheries (Fiji Software, NIH). Only single cells were taken into account. The migration speed of cells was determined with time-lapse microscopy over a period of at least 10 h in 5 min intervals. A Tokai-Hit Imaging Chamber (Tokai Hit) that maintained a humid 37 °C and 5% CO_2_ environment was first equilibrated for 1 h. Cell cultures were placed inside the chamber mounted on a DMIRE2 inverted microscope (Leica) equipped with an ASI *x*/*y*/*z* stage (BioVision Technologies) and a Hamamatsu camera; a 10× air lens was used for image sequence recording. Cell migration speed *υ* (length of the total trajectory *d* divided by time *t*) was calculated by tracing the (*x*,*y*) position of the center of the cell nucleus at every image using ImageJ Software (NIH) and the Manual Tracking plugin (https://imagej.nih.gov/ij/).

### Immunofluorescence

Cells were fixed with 4% (w/v) paraformaldehyde (Sigma) for 10 min at room temperature, permeabilized with 0.1% (v/v) Triton X-100 in Tris buffered saline (TBS) for 10 min, and then incubated for 1 h with primary antibodies directed against vinculin (1:1000; mouse monoclonal anti-vinculin IgG; sc-73614 Santa Cruz). In the next step the cells were incubated with secondary antibodies: 1:1000; Alexa-Fluo 568 goat anti-mouse IgG; A-11004 (Invitrogen), phalloidin–tetramethylrhodamine B isothiocyanate (1 μg/ml; Sigma) and DAPI (1 μg/ml; Sigma).

### Traction force microscopy

Cell traction force was determined by first measuring the hydrogel displacement field and then solving the inverse elastic problem to reconstitute the traction force map. Specifically, red fluorescent beads (200 nm, Thermo Fisher Scientific) were mixed at a density of 0.3% (v/v) with the 30 kPa PAAm pre-gel solution containing 10.4% (w/v) acrylamide and 0.264% (w/v) bis-acrylamide. After polymerization, hydrogels were activated with 0.2 mg/ml Sulfo-SANPAH and then coated with 0.1 mg/ml vimentin (62). hMSCs were sparsely seeded on the vimentin-coated hydrogels to minimize the cell–cell contact. A bright field image of a randomly selected cell and the corresponding fluorescent beads image was taken. The cell was then detached from the hydrogel with 0.5 N sodium hydroxide, and the reference image of the fluorescent beads was taken. The in-plane displacement field of the hydrogel substrate was calculated by particle image velocimetry. The traction force field was then constructed by solving the inverse problem of Boussinesq solution through Fourier transform traction force cytometry (63, 64).

### 3D spheroid formation and analysis

To perform cell aggregate invasion assays, cell aggregates were first generated using the procedure described by Ibidi (https://ibidi.com/img/cms/support/AN/AN32_Generation_of_spheroids.pdf). Briefly, 96-well flat-bottom plates were coated with 40 μl of 1% (w/v) agarose solution diluted with PBS. Wildtype and vimentin-null mEF were then plated at 30,000 cells/well in 100 μl of DMEM (DMEM/F12, 10% FBS, 1xPen/Strep). Cell aggregates were incubated at 37 °C with 5% CO_2_ for 2 days before harvesting. The diameter of the spheroids was between 100 and 200 μm when collected. Collagen gels were prepared using a slightly modified protocol from Ibidi (https://ibidi.com/img/cms/support/AN/AN26_CollagenI_protocols.pdf). 5 mg/ml rat tail collagen type I (Ibidi) was diluted on ice to 1.5 mg/ml with 6.67% (v/v) of 5× DMEM (Fisher scientific) and 10% FBS in 1x DMEM. The pH was adjusted to 7.4 using 1 M NaOH and sodium bicarbonate. Cell aggregates were added to the collagen solution before it solidified. The collagen solution was then pipetted onto 1% agarose-coated 24-well flat bottom plates and allowed to polymerize for 40 min at 37 °C, 5% CO_2_, and 100% humidity. To assess the aggregates’ invasion through the collagen network alone or with the presence of either native vimentin or citrullinated vimentin, 2 μg/ml of each type of vimentin was added to the collagen/cell mixture before polymerization. Citrullinated vimentin in 11.5 mM Tris, 0.2 mM CaCl_2_, 6.1 mM DTT, and 0.9 mM EDTA, pH 7.5, was diluted 4× in PBS, pH 7.4 and then diluted 450× in the collagen gel media to a concentration of 2 μg/ml. Uncitrullinated vimentin (1 mg/ml in 1 mM DTT and 5 mM Tris, pH 7.5) was diluted 400× into the collagen gel medium to a concentration of 2 μg/ml. DMEM complete medium (500 μl) was added on top of the fully polymerized collagen gel (440 μl). After 3 h inside the incubator, spheroids in collagen gel were imaged for 48 h with phase-contrast widefield using a Plan Fluor 10× objective on Nikon Eclipse Ti inverted microscope equipped with an Andor Technologies iXon em+ EMCCD camera (Andor Technologies). Aggregates were maintained at 37 °C and 5% CO2 using a Tokai Hit (Tokai-Hit) stage top incubator. Cross-sectional area of the spheroid (core and connecting strands) was traced using ImageJ.

## Data availability

The manuscript contains all data described within the text.

## Supporting information

This article contains supporting information.

## Conflict of interest

The authors declare no conflict of interest with the contents of this article.

## Supporting information

Supporting Figure S4 **Analysis of vimentin-null cell expansion through collagen matrices with and without extracellular vimentin and its citrullinated form.** Representative phase-contrast images of cell aggregates formed by vimentin-null mEF embedded in matrixes comprised of (*A*) collagen I, (*B*) collagen I + vimentin, and (*C*) collagen I + citrullinated vimentin at 24 h. *D*, averaged projected area of cell aggregates formed from vimentin-null mEF embedded in collagen network with vimentin or extracellular vimentin (2 μg/ml). mEF, mouse embryonic fibroblast.
