# Supplementary material for: Extracellular vimentin is sufficient to promote cell attachment, spreading, and motility by a mechanism involving N-acetyl glucosamine-containing structures
Source: J Biol Chem. 2023 Jun 24;299(8):104963. doi: 10.1016/j.jbc.2023.104963 (PMC10392088; doi:10.1016/j.jbc.2023.104963)
Supplement: Supporting Figure S4 — Analysis of vimentin-null cell expansion through collagen matrices with and without extracellular vimentin and its citrullinated form. Representative phase-contrast images of cell aggregates formed by vimentin-null mEF embedded in matrixes comprised of (A) collagen I, (B) collagen I + vimentin, and (C) collagen I + citrullinated vimentin at 24 h. D, averaged projected area of cell aggregates formed from vimentin-null mEF embedded in collagen network with vimentin or extracellular vimentin (2 μg/ml). mEF, mouse embryonic fibroblast. [file mmc1.pdf]

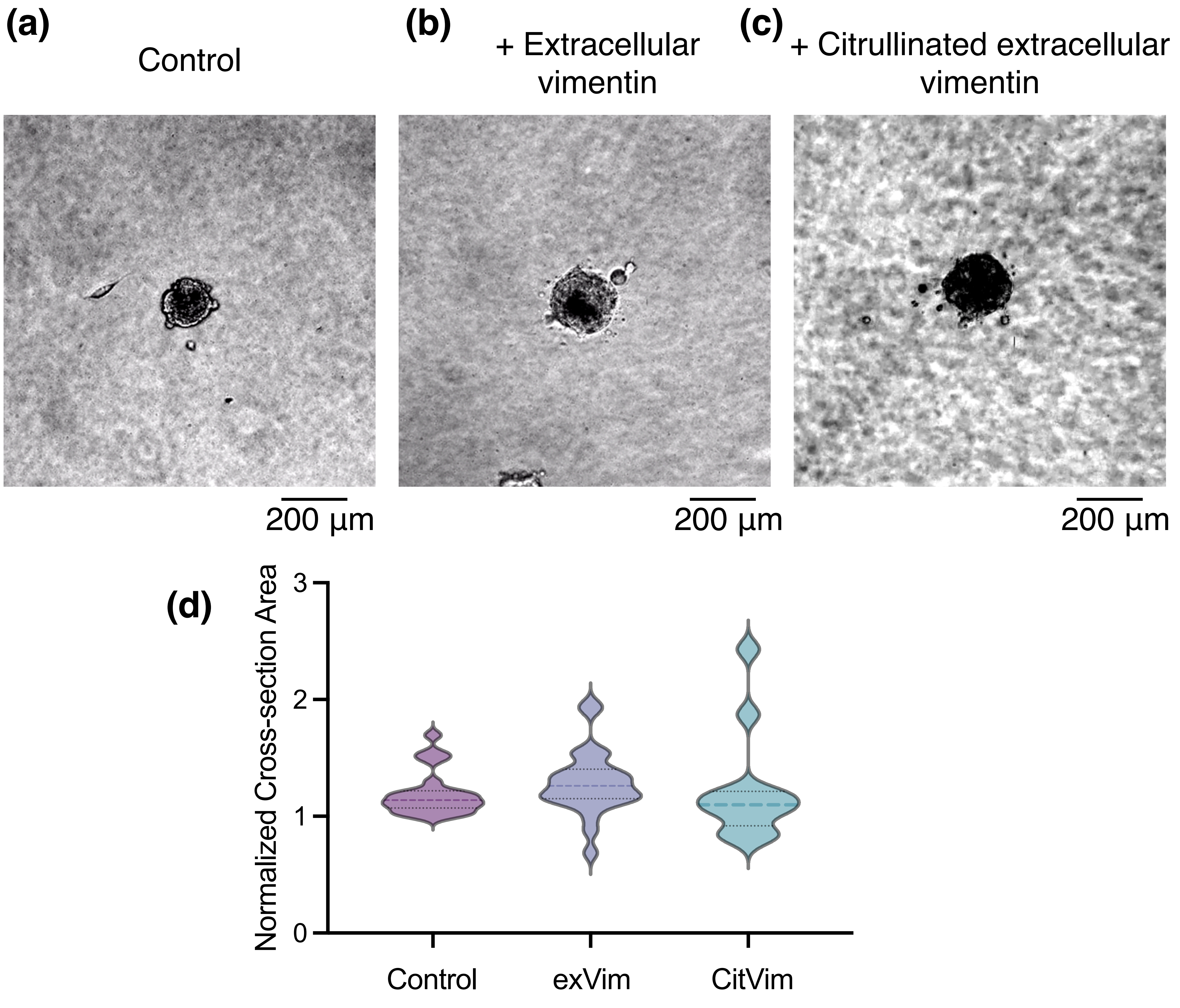
(a)
Control
(b)
+ Extracellular vimentin
(c)
+ Citrullinated extracellular vimentin
200 μm
200 μm
200 μm
(d)
Normalized Cross-section Area
3
2
1
0
Control
exVim
CitVim
